# Dileucine-supplemented essential amino acids support whole-body anabolism after resistance exercise and serum-stimulated cell-based anabolism

**DOI:** 10.1080/15502783.2025.2590090

**Published:** 2025-11-30

**Authors:** Jonathan A. Aguilera, Cassidy T. Tinline-Goodfellow, Matthew J. Lees, Ines Kortebi, Daniel W. D. West, Sidney Abou Sawan, Megha Sharma, Raza Bashir, Takeshi M. Barnes, Alexander V. Ulanov, Nicholas A. Burd, Daniel R. Moore

**Affiliations:** a Faculty of Kinesiology and Physical Education, University of Toronto, Toronto, ON, Canada; b KITE Research Institute, Toronto Rehabilitation Institute, Toronto, ON, Canada; c Iovate Health Sciences International Inc, Oakville, ON, Canada; d Department of Health and Kinesiology, University of Illinois Urbana-Champaign, Urbana, IL, USA; e Roy J. Carver Biotechnology Center, University of Illinois Urbana-Champaign, Urbana, IL, USA; f Division of Nutritional Sciences, University of Illinois Urbana-Champaign, Urbana, IL, USA

**Keywords:** Dileucine, essential amino acids, supplementation, resistance training, whole-body anabolism

## Abstract

**Background:**

Essential (EAA) and branched chain (BCAA) amino acid ingestion support whole-body anabolism after resistance exercise and can attenuate markers of postexercise myofibrillar protein breakdown (i.e. urinary 3-methylhistidine; 3MH). Leucine is often considered a primary anabolic EAA through its ability to activate the mechanistic target of rapamycin complex 1 (mTORC1) and stimulate muscle protein synthesis. The dipeptide leucine (dileucine) has been shown to more effectively stimulate myofibrillar protein synthesis than leucine in young males at rest. Therefore, we aimed to determine the effect of a dileucine-containing essential amino acid formula (DIEAA; 2 g dileucine, 1 g leucine, 9.15 g total EAA) on the anabolic and catabolic responses following resistance exercise in young recreationally active adults when compared with ingesting branched chain amino acids (BCAA; 3 g leucine, 1.5 g isoleucine, 1.5 g valine) or isonitrogenous (to DIEAA) collagen hydrolysate (COL).

**Methods:**

In a randomized, double-blind, crossover design, 12 healthy adults (8 M, 4F, aged 24 ± 3 y) performed a 60 min bout of whole-body resistance exercise, after which they ingested DIEAA, BCAA, or COL protein beverages containing 100 mg L-[1-^13^C]leucine (#NCT05754125). Total exogenous leucine retention (as an estimate of whole-body anabolism) was assessed over the 6 h postprandial period by determining total leucine oxidation from ^13^CO_2_ enrichment (isotope ratio mass spectrometry) in repeated breath samples. A urinary 3MH:creatinine ratio (3MH:Cr) over 6 h was used as an estimate of skeletal muscle myofibrillar protein breakdown. To further assess the anabolic potential of nutrients, C2C12 myotubes were treated with a subset (*n* = 7) of human serum-conditioned media for 4 h to measure downstream mTORC1 substrate phosphorylation, protein synthesis (puromycin and L-*ring*-[D_5_]phenylalanine incorporation) and breakdown (ubiquitinated protein), and myotube hypertrophy.

**Results:**

Total exogenous leucine retention were similar (*p* = 0.68) between DIEAA (215.72 ± 42.45 μmol·kg^−1^) and BCAA conditions (219.15 ± 45.26 μmol·kg^−1^), with both DIEAA and BCAA being greater (*p* < 0.0001) than COL (37.25 ± 8.16 μmol·kg^−1^). There were no differences (*p* = 0.58) in 3MH:Cr between supplement conditions. There was no effect of condition *ex vivo* on puromycin incorporation into nascent peptides (*p* = 0.31), total protein ubiquitination as an estimate of protein breakdown (*p* = 0.59), phosphorylation of downstream mTORC1 substrates *p*-RPS6^S240/244^ (*p* = 0.39) or *p*-4E-BP1^T37/46^ (*p* = 0.50), and myotube diameter (*p* = 0.55). Stable isotope-derived rates of mixed muscle protein synthesis (MPS) demonstrated a trend toward a main effect (*p* = 0.086) with pairwise comparisons revealing a large effect of DIEAA compared to COL (dz = 1.47), a medium effect of DIEAA compared to BCAA (dz = 0.81), and a trivial effect of BCAA comapred to COL (dz = 0.002).

**Conclusions:**

Dileucine-supplemented EAA and BCAA support greater whole-body anabolism compared with COL after resistance exercise independent of attenuation in urinary estimates of myofibrillar protein breakdown. Exploratory *ex vivo* experiments reveal a potential anabolic effect of DIEAA in stimulating MPS. Collectively, these findings suggest that consuming dileucine with sufficient EAA and BCAA increases exogenous leucine retention to support whole-body anabolism during postexercise recovery in individuals performing resistance training.

## 1 Introduction

Dietary amino acids provide substrates for the synthesis of muscle and whole-body proteins [1–3]. The postexercise consumption of essential amino acids (EAA) has long been known to support a positive net muscle and whole-body net protein balance through their ability to support greater rates of muscle protein synthesis (MPS) and attenuate the exercise-induced stimulation of muscle protein breakdown [4,5]. Branched-chain amino acids (BCAAs), especially leucine, have been shown to be potent stimulators of the mechanistic target of rapamycin complex 1 (mTORC1) pathway [6,7] and the MPS in humans at rest [8,9] and after resistance exercise [10,11]. We have previously shown that the anabolic effects of EAA and BCAA are evident at the whole-body level, with their postexercise ingestion resulting in greater utilization of dietary leucine for the synthesis of whole-body proteins to support postexercise recovery while attenuating markers of myofibrillar catabolism [12]. Interestingly, it was recently demonstrated that the ingestion of the di-peptide dileucine elevated plasma leucine and dileucine concentrations and stimulated a greater increase in resting myofibrillar protein synthesis than did an equivalent amount of leucine [13], suggesting a potential role for bioactive peptides in the regulation of protein synthesis in humans. However, the ability of dileucine to support postexercise anabolism has yet to be determined in humans.

The measurement of muscle protein breakdown is technically and logistically challenging during the postprandial period and can be prohibitively invasive [14]. Thus, less is known about the nutritional regulation of muscle protein breakdown in humans during the postexercise recovery period. However, the methylation of histidine is a posttranslational modification of this amino acid for mature actin/myosin protein, generating 3-methylhistidine (3MH), which has been used historically [15] and recently [16] as a biomarker for myofibrillar protein breakdown. We [12] and others [17,18] have shown that EAA ingestion after exercise can lower urinary 3MH levels, suggesting attenuation of myofibrillar protein breakdown. Alternatively, providing only BCAA did not attenuate postexercise 3MH [12], suggesting that the previously observed BCAA-induced stimulation of muscle protein synthesis may need to be supported by a reciprocal increase in muscle protein breakdown to provide the necessary amino acid precursors, as previously suggested [19]. Therefore, nutritional strategies that enhance anabolism and attenuate (muscle) catabolism may represent effective means to support recovery from resistance exercise.

To further examine the mechanisms of MPS without the invasive use of muscle biopsies, an in vitro model using *ex vivo* human serum has been developed [20]. As human serum reflects the integrated systemic response to different nutritional and exercise conditions, it has been proposed that coculturing myotubes with *ex vivo* human serum may increase the physiological relevance of cell culture models [21]. Previous work has demonstrated that media conditioned with whey protein hydrolysate-fed serum elicited greater MPS and anabolic signaling compared to isonitrogenous non-essential amino acid-fed serum did [22], highlighting the ability of the *ex vivo* model to differentiate anabolic responses between dietary protein formulations that have been shown *in vivo* [23]. Therefore, by collecting blood samples in the postabsorptive and postprandial states, the *ex vivo* cell model may elucidate the mechanisms by which dileucine ingestion can support muscle anabolism compared with an equal amount of leucine ingestion [13].

The overarching objective of the current study was to investigate the anabolic and catabolic response to the ingestion of a dileucine-containing EAA formula (DIEAA) compared with branched-chain amino acid (BCAA) and an isonitrogenous nonessential amino acid collagen (COL) control in young healthy adults. Based on our previous observation of an attenuation in 3MH with EAA compared to BCAA ingestion after exercise [12], our primary objective was to determine whether an EAA formulation enriched with dileucine reduced urinary 3MH compared to BCAA and COL. Furthermore, using our noninvasive oral leucine tracer model [24], we aimed to determine whether leucine retention (as a proxy for whole-body protein synthesis) was enhanced by the ingestion of EAA in DIEAA or BCAA compared to a primarily nonessential amino acid-based COL protein control. Finally, as an exploratory arm, we aimed to determine the impact of postexercise amino acid ingestion on intracellular signaling, muscle protein synthesis and breakdown, and hypertrophy in C2C12 myotubes conditioned with postprandial serum *ex vivo*. We hypothesized that urinary 3MH would be lowest in DIEAA and that leucine retention would be greater in DIEAA compared with BCAA and COL. We also hypothesized that the *ex vivo* cell model would show enhanced anabolic signaling, puromycin incorporation, and cellular hypertrophy in DIEAA compared with BCAA and COL.

## 2 Methods

### 2.1 Participants and ethics approval

Twelve healthy adults (8 M, 4F; Table 1) provided informed written consent after being informed of the study purpose, protocols, and risks. Participants were recruited through postings at the University of Toronto and were deemed eligible to participate if they were (i) recreationally active (i.e. performed structured exercise ≥2× per week for the previous 6 months); (ii) considered physically fit to perform strength tests based on the responses to a Physical Activity Readiness Questionnaire [25] and an International Physical Activity Questionnaire [26]; and (iii) not taking oral contraceptives. Individuals were excluded if they were (i) currently using tobacco products; (ii) currently using or had a history of anabolic steroid use; (iii) unable to abstain from supplement use for ≥3 weeks prior to metabolic trials (e.g. creatine, BCAA); (iv) diagnosed with a medical condition (e.g. type 2 diabetes, heart disease, and cancer); (v) amenorrheic (females only); and (vi) currently using medications known to affect protein metabolism (e.g. statins, prescription anti-inflammatories). The study protocol was performed in accordance with the Declaration of Helsinki and approved by the University of Toronto Research Ethics Board (#37843). The present study was registered as a clinical trial at ClinicalTrials.gov (#NCT05754125).

**Table 1.** Participant characteristics.

Characteristics	Mean ± SD
Age (years)	27.0 ± 4.7
Height (cm)	172.0 ± 9.8
Body mass (kg)	72.0 ± 17.5
Fat-free mass (kg)	58.3 ± 11.7
Body fat (%)	18.3 ± 6.7
Habitual protein intake (g·kg^−1^·d^−1^)	1.7 ± 0.7
Habitual energy intake (kcal·d^−1^)	2103 ± 478

### 2.2 Experimental design

The present study used a double-blind, placebo-controlled, randomized crossover design. All testing procedures and data collection took place at the Goldring Center for High Performance Sport at the University of Toronto. The participants reported to the laboratory ≥4 days prior to the first metabolic trial for baseline testing, which included a body composition assessment and exercise familiarization. The participants were instructed to arrive after a 10-h overnight fast and to avoid ingesting fluids on the morning of the body composition assessment. Participants' fat-free mass was estimated using the Brozek equation [27] by measuring body density via air displacement plethysmography (BOD POD, Cosmed USA Inc., Concord, CA, USA) and body mass with a calibrated weight scale. Following the body composition assessment, participants were provided a carbohydrate beverage containing 1 g CHO·kg^−1^ body weight (1:1 ratio of maltodextrin and Gatorade powder; PepsiCo, Inc., Harrison, NY, USA) and allowed time to rehydrate (~30 min). The participants were then familiarized with the whole-body resistance exercise protocol and guided through one-repetition maximum (1RM) testing as previously described [28].

### 2.3 Metabolic trial

Prior to the first metabolic trial, the participants were instructed to record their habitual dietary intake for 72 h using MyFitnessPal (San Francisco, CA, USA). The participants were then instructed to replicate the final 24 h of the diet log on the day before the second and third metabolic trials. The participants were prohibited from engaging in structured physical activity and alcohol for 48 and 24 h, respectively, prior to each trial. Upon arrival, after an overnight fast, baseline (*t* = −60 min) breath and urine samples were collected in 10 mL Vacutainers (BD, Franklin Lakes, NJ, USA) and spot urine containers, respectively, before participants began the whole-body bout of resistance exercise (described below). An additional baseline breath sample was collected halfway (*t* = −30 min) through the exercise protocol. Immediately following resistance exercise, an intravenous catheter was inserted into an antecubital vein by a trained phlebotomist, and two baseline serum samples were collected into 10 mL serum separator tube incubators (BD; #367988), alongside a final baseline breath sample. Subsequently, participants ingested the trial supplement (*t* = 0 min), and breath samples were collected every 20 min for the first 3 h and every 30 min thereafter over a 6-h measurement period. This period was selected as it corresponds with the expected peak concentrations of dileucine and EAA [11,13,24]. During this 6-h period, all urine excreted by participants was collected into a 2 L container from which a representative sample was aliquoted and stored at −80 °C until analysis. Additionally, serum samples were collected at 15 and 30 min of the measurement period for *ex vivo* screening. Serum samples were allowed to clot at room temperature for 30 min and then centrifuged at 1500 × g for 15 min at 4 °C, aliquoted, and stored at −80 °C until further analysis.

### 2.4 Resistance exercise protocol

The exercise protocol was designed to target all major muscle groups and has been previously shown to support whole-body anabolism and reduce exercise performance during recovery [29]. The protocol included upper body supersets (two exercises performed consecutively with no dedicated rest between the two exercises) and isolated lower body exercises as follows: 1) bench press and cable row superset; 2) shoulder press and latissimus pulldown supersets; 3) leg press; and 4) knee extension. Following a 5-min warm-up on a cycle ergometer, participants completed four working sets of 8−12 repetitions at 75% 1RM for each exercise with ~90 s of rest between sets. During rest, the participants were asked to provide their rating of perceived exertion (RPE) for the previous set using the Borg CR10 scale [30]. The investigator adjusted the weight throughout the session to ensure that the 8−12 repetition range was met while achieving a high RPE (≥7), close to volitional failure.

### 2.5 Trial supplements

All three supplements were prepared in powder form and provided by Iovate Health Sciences International (Iovate Health Sciences Inc., Oakville, ON, Canada). The supplement order for each participant was determined by block randomization, and the supplements were provided in a double-blinded fashion. The dileucine-containing EAA supplement (DIEAA) used in the present study contained a total of 9.15 g of EAA, 2 g of which was dileucine and 1 g of which was leucine. The 2 g dileucine was selected because it has previously been shown to support resting myofibrillar protein synthesis [13] as well as training-induced increases in muscle strength [31] to a greater extent than 2 g of leucine does. The additional leucine and EAA doses were provided to approximate the content of 20–25 g of whey protein, which enhances postexercise muscle protein synthesis [32,33]. The branched-chain amino acid (BCAA) supplement consisted of 3 g of leucine, 1.5 g of isoleucine and 1.5 g of valine to be equivalent to the BCAA content of DIEAA. The placebo supplement (COL) consisted of collagen protein and was designed to be nitrogen-matched to DIEAA but to provide low-EAA control, which would have little effect on muscle protein synthesis [22,23]. All the supplements were enriched by the addition of 100 mg of 99% [1-^13^C]leucine (Cambridge Isotope Laboratories Inc., Tewksbury, MA, USA) to measure exogenous leucine oxidation at each time point.

### 2.6 Analysis of urine samples

The urinary 3MH concentration was measured with a commercially available ELISA kit (MyBioSource, San Diego, CA, USA). The urine samples were subsequently centrifuged (1000 × g for 20 min at 4 °C). To remove insoluble debris, the supernatant was used for the analysis. To account for sample dilution due to hydration status, urinary 3MH measures were normalized to urinary creatinine [34,35], as measured by a QuantiChrom Creatinine Assay Kit (BioAssay Systems, Hayward, CA, USA).

### 2.7 Analysis of breath samples

Breath samples were collected into sterile 10 mL vacutainers and stored at room temperature before analysis of ^13^CO_2_ enrichment by isotope ratio mass spectrometry (Compact Science Systems, Newcastle, UK), as previously described [24].

### 2.8 Calculations

Exogenous leucine oxidation (Exo Ox) was calculated as previously described [24], with the exception that a correction factor for the retention of ^13^C in the bicarbonate pool was not applied. Total exogenous leucine oxidation (in μmol/kg) over the 6-h postprandial period was calculated using the trapezoidal area under the curve (AUC) of Exo Ox divided by the change in time. As a marker of anabolic sensitivity, total exogenous leucine retention (in μmol/kg) was calculated as the difference between leucine intake and total exogenous leucine oxidation.

### 2.9 Serum amino acid concentrations

Serum amino acid concentrations for a subset of participants whose serum was used for *ex vivo* FSR experiments were determined by liquid chromatography-tandem mass spectrometry (LC-MS/MS; Thermo Altis Triple Quadrupole, Thermo Fisher Scientific, Waltham, MA) as described previously [36]. For each participant, a fasted serum sample was pooled from all three metabolic trials, and three postprandial samples (one for each metabolic trial) were combined with 15- and 30-min serum.

### 2.10 *Ex vivo* experiments

C2C12 myoblasts (CRL−1772) were obtained from the American Type Culture Collection and expanded in growth media consisting of high-glucose Dulbecco's modified Eagle's medium (DMEM; #D5796--500ML, Sigma-Aldrich) supplemented with 10% fetal bovine serum (FBS; #F1051--500ML, Sigma-Aldrich) and 1% penicillin-streptomycin (PS; #15140122, Gibco) at 37 °C with 5% CO_2_ in a humidified environment. Prior to the experiments, the cells were seeded into 6-well tissue culture plates in growth media. Once cells reached >90% confluence, the medium was changed to differentiation media comprised of low-glucose DMEM (#D6046-500ML, Sigma-Aldrich) supplemented with 2% horse serum (#16050122, Gibco) and 1% PS to induce differentiation into myotubes for 5 days. The differentiation media was changed every 48 h, and all experiments were performed prior to passage 10.

After 5 days of differentiation, 24 h after the most recent media were changed, the myotubes were washed once in Dulbecco's phosphate-buffered saline (DPBS; #D8537--500ML, Sigma-Aldrich) and then starved of amino acids and serum in amino acid-free DMEM (#D9800−13, United States Biological) supplemented with 3.7 g/L sodium bicarbonate (#SOB308, BioShop), 1 mM sodium pyruvate (#11360070; Gibco), and 1% penicillin-streptomycin (#2503008; Gibco) for 1 h [37,38]. Following starvation, the cells were refed in amino acid-free DMEM supplemented with 20% (v/v) human serum, pooled with fasted serum from all three metabolic trials, or combined with 15 and 30 min of postprandial serum from each metabolic trial (i.e. 1 starvation and 3 feeding conditions per participant) for 4 h at 37 °C [39]. Puromycin (#PUR333, BioShop) was added dropwise to a concentration of 1 µM for the final 30 min of the refeeding period. At 4 h of refeeding, the plates were placed on an ice block, rinsed twice in ice-cold DPBS and collected in ice-cold RIPA buffer (50 mM Tris-HCl pH 7.5; 150 mM NaCl; 1% Triton-X100; 0.5% sodium deoxycholate; 0.1% sodium dodecyl sulfate) supplemented with a 1x protease inhibitor tablet (#11836170001, Roche) and a 1x phosphatase inhibitor tablet (#A32957, Thermo-Scientific) per 10 mL of RIPA buffer. Two wells per condition were pooled, and experiments were repeated in duplicate on separate passages. Lysates were kept on ice for 1 h and then snap-frozen in liquid nitrogen and stored at −80 °C until processing.

In a separate single experiment using the same conditions, 200 μM L-[*ring*-^2^H_5_]phenylalanine (#DLM−1258, Cambridge Isotopes) was added to the refeeding media from a 10 mM stock in DPBS to measure protein synthesis (described below) over the course of the 4 h serum stimulation period. Two wells per condition were pooled, and the lysates were kept on ice for 1 h and then snap-frozen in liquid nitrogen and stored at −80 °C until processing (described in “Protein Synthesis Analysis” below).

### 2.11 Myotube diameter experiments

C2C12 myoblasts were seeded into 24-well plates and allowed to proliferate until >90% confluent prior to being differentiated in low glucose differentiation medium. On day 5 of differentiation, the myotubes were starved of amino acids and serum and refed for 4 h, as described above. Following refeeding, the myotubes were washed 1× in ice-cold DPBS and fixed in 4% paraformaldehyde for 15 min at room temperature (RT). Fixed cells were rinsed twice in DPBS, permeabilized and blocked in 0.3 M glycine/0.3% Triton X−100/5% normal goat serum (#16210064, Gibco) diluted in DPBS for 60 min. The myotubes were then incubated with anti-Desmin primary antibody (1:100, #D8281, Sigma-Aldrich) in 0.3% Triton X−100/1% bovine serum albumin (BSA) in DPBS for 1 h at RT. The myotubes were subsequently rinsed twice in DPBS and incubated with secondary antibody (1:300; Alexa Fluor 488 goat anti-rabbit IgG H + L; #A−11008, Invitrogen) in 1% BSA/DPBS in the dark. The cells were then rinsed once in DPBS and incubated with DAPI (1:5,000 in PBS; #4083; Cell Signaling Technology) for 5 min in the dark. Finally, the cells were rinsed twice in DPBS, stored in DPBS at 4 °C in the dark overnight and imaged the next day. Images of at least five random fields per condition were taken at 10× magnification using an EVOS FL Auto Cell imaging microscope (Thermo Fisher, Waltham, MA). Myotube images were analyzed using ImageJ FIJI (US National Institutes of Health, Bethesda, MD), where the means of three measurements along the length of each myotube were used to calculate diameter, and an over 100 fibers per condition (range: 100−108 fibers) were analyzed [37,39].

### 2.12 Immunoblotting

The cell lysates were homogenized on ice using a polytron for 30 s, and then clarified via centrifugation at 8000 g for 15 min at 4 °C. The protein concentrations of the supernatants were determined by bicinchoninic acid (BCA) assay (#23227, Thermo Scientific), and the lysates were diluted to equal concentrations with 4X Laemmli sample buffer and RIPA buffer prior to being heated at 95 °C for 5 min. Equal amounts of protein (20 μg) from each sample were loaded onto precast 8%−16% gradient Criterion™ TGX Stain-Free™ gels (#5678105, Bio-Rad) and separated by SDS‒PAGE for ~40 min at 200 V. Protein gels were imaged using Bio-Rad Stain-Free™ technology (45 s activation) as a loading control for normalization to total protein. Proteins were then transferred at 100 V for 1 h onto 0.45 µm nitrocellulose membranes (#1620115, Bio-Rad) using a wet transfer. The membranes were then blocked in 5% skim milk in Tris-buffered saline with 0.1% Tween−20 (TBST) for 1 h at RT prior to incubation with primary antibodies overnight at 4 °C. The primary antibodies were all diluted 1:1000 in TBST with 5% BSA and were purchased from Cell Signaling Technology (CST; Danvers, MA), unless stated otherwise. Antibodies consisted of *P*-RPS6^Ser240/244^ (#5364), *p*-4E-BP1^Thr37/46^ (#2855), anti-ubiquitin (#3936), and anti-puromycin (1:2000; #MABE343, Millipore). The next day, the membranes were washed in TBST (3 × 5 min) and then incubated with either anti-rabbit (#7074) or anti-mouse (#7076) HRP-conjugated antibodies (both 1:10,000 in TBST) for 1 h at RT. The membranes were then washed in TBST (3 × 5 min), and with Clarity™ Western ECL substrate (#1705061, Bio-Rad), they were imaged using a Bio-Rad ChemiDoc imaging system. Bands were quantified using ImageLab software (Bio-Rad), and the Stain-Free image was used to control for potential differences in total protein between lanes.

### 2.13 Protein synthesis analysis

Lysates from cells treated with L-[*ring*-^2^H_5_]phenylalanine were homogenized with a polytron on ice for 30 s. Proteins were precipitated with 1 M PCA and centrifuged at 1500 × g for 15 min. The resulting protein pellet was gently rinsed once in ice-cold 70% ethanol before centrifugation at 1500 g for 15 min, and the ethanol supernatant was discarded. Subsequently, 750 μL of 0.1 M HCl and 750 μL of Dowex resin (#Dowex50W-X8–200; Sigma-Aldrich) in 1 M HCl were added to the protein pellet, and the proteins were hydrolyzed for 36 h at 110 °C before being purified over cation exchange columns constructed from glass wool in 5 mL syringes. Purified amino acids were dried down under a steady stream of nitrogen at 80 °C, resuspended in 0.1% Formic acid, and analyzed at the Analytic Facility for Bioactive Molecules at SickKids (Toronto, Canada) by liquid chromatography tandem mass spectrometry (LC-MS/MS) monitored at mass to charge ratios (m/z) of 171.1/125 for L-[*ring*-^2^H_5_]phenylalanine and 166.1/131 for natural phenylalanine, as previously described for human skeletal muscle [40].

Fractional synthetic rates (FSR) for a mixed fraction were calculated as previously described [41] using the equation:

$$\mathrm{FSR}=(\frac{E_{\mathrm{incorp}}}{E_{\mathrm{Medium}}}\times t^{-1})\times 100$$

where E_incorp_ is the enrichment of protein-bound L-[*ring*-^2^H_5_]phenylalanine, E_medium_ is the enrichment of the media calculated from serum phenylalanine concentrations (above), adjusted to 20% to account for the volume of serum used during the refeeding period, and *t* is time (4 h). The FSR is expressed as a percentage per hour.

### 2.14 Statistical analysis

The primary outcome for the present study was urinary 3MH:Cr (3-methylhistidine to creatinine ratio). An *a priori* power analysis, with *α* = 0.05 and 1-*β* = 0.80, was performed using previous methods [12], which revealed that a sample size of 12 is sufficient to detect a 23% lower postexercise 3MH:Cr concentration following the ingestion of EAA compared with BCAA. The data were analyzed via GraphPad Prism (version 9.3.0, GraphPad Software, San Diego, CA, USA), with significance set at *P* < 0.05. Values were classified as outliers if they exceeded both two standard deviations from the group mean and the 1.5 × IQR bounds. One participant whose serum dileucine concentration met both criteria was excluded from the serum dileucine analysis. The final sample size for serum dileucine analysis was *n* = 6, as indicated in the figure legend. Urinary 3MH:Cr, serum amino acid concentration AUC, total exogenous leucine oxidation, and total exogenous leucine retention for all conditions were analyzed by one-way repeated measures ANOVA, with Tukey's post hoc correction for multiple comparisons used to identify differences between conditions when there was a main effect. The Exo Ox for all conditions was analyzed by two-way repeated measures ANOVA (condition × time). Where sphericity was violated, a Greenhouse–Geisser correction was applied to all main effects and interactions. Where significant effects were identified in the ANOVA, Tukey's post hoc adjustment was performed to determine differences between conditions. Serum from a subset of 7−10 participants was used for *ex vivo* experiments depending on serum availability and sample hemolysis (see figure descriptions for exact sample sizes). This sample size is in line with previous work using the *ex vivo* model [20,22,37–39]. *Ex vivo* outcomes were analyzed by one-way repeated measures ANOVA, with post hoc pairwise comparisons using Tukey's HSD correction. Effect size was calculated as Cohen's dz (mean of paired differences divided by the standard deviation of those differences), with thresholds of <0.2 for trivial, 0.2–0.6 for small, 0.6–1.2 for medium, and >1.2 for large. *P*-values are presented as corrected, exact values, unless less than 0.001. The data are reported as mean ± SD, unless stated otherwise.

## 3 Results

### 3.1 Urinary 3-methylhistidine

**Figure 1.**
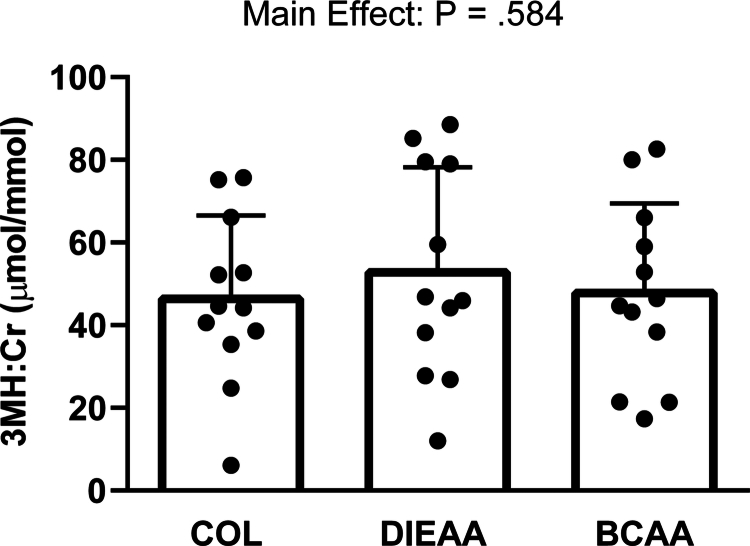
Pooled 8 h urinary 3-methylhistidine (3MH) and creatinine (Cr) were measured via ELISA for each trial. There was no main effect of supplement condition (*P* = 0.584). Data are expressed as the 3MH:Cr ratio and presented as the means ± SD. COL, Collagen; DIEAA, Dileucine-containing essential amino acid formula; BCAA, Branched-chain amino acids.

There were no differences in urinary 3MH:Cr across the supplement conditions (*P* = 0.584; Figure 1).

### 3.2 Serum amino acid concentrations

**Figure 2.**
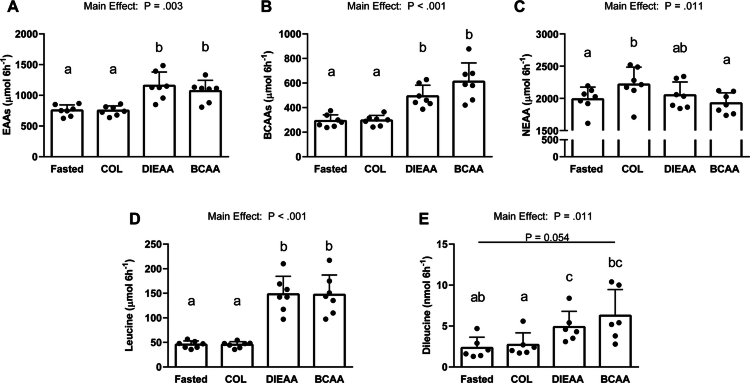
Serum concentrations of essential amino acids (A; *n =* 7), branched-chain amino acids (B; *n =* 7), nonessential amino acids (C; *n* = 7), leucine (D; *n =* 7), and dileucine (E; *n =* 6), expressed as the means ± SD. Data are presented for the fasted state (pooled from all three metabolic trials) and for the combined 15- and 30-min postprandial serum samples from each metabolic trial (COL, DIEAA, BCAA). EAA main effect, *P* = 0.003. BCAA main effect, *P* < 0.001. NEAA main effect, *P* = 0.011. Leucine main effect, *P* < 0.001. Dileucine main effect, *P* = 0.011. Conditions that do not share a letter are significantly different (*P* < 0.05). COL, Collagen; DIEAA, Dileucine-containing essential amino acid formula; BCAA, Branched-chain amino acids.

There was a main effect of the supplement condition on serum concentrations of EAA (*P* = 0.003), BCAA (*P* < 0.001), non-EAA (*P* = 0.011), and leucine (*P* < 0.001). Tukey's post hoc testing revealed that serum concentrations of EAA, BCAA, and leucine were significantly greater in the DIEAA and BCAA conditions compared to both COL and fasted levels, with no differences observed between DIEAA and BCAA (Figure 2).

For the serum dileucine concentrations, there was a trend towards a main effect of supplement condition (*P* = 0.079). However, after removing a potential outlier (*n* = 6), the main effect was statistically significant (*P* = 0.011). As shown in panel E of Figure 2, post hoc testing revealed that dileucine concentrations were significantly greater in the DIEAA condition compared to fasted (*P* = 0.003) and COL (*P* = 0.002) conditions. Furthermore, the dileucine concentrations in the BCAA condition were significantly greater compared to COL condition (*P* = 0.047).

### 3.3 ^13^CO_2_ breath test

**Figure 3.**
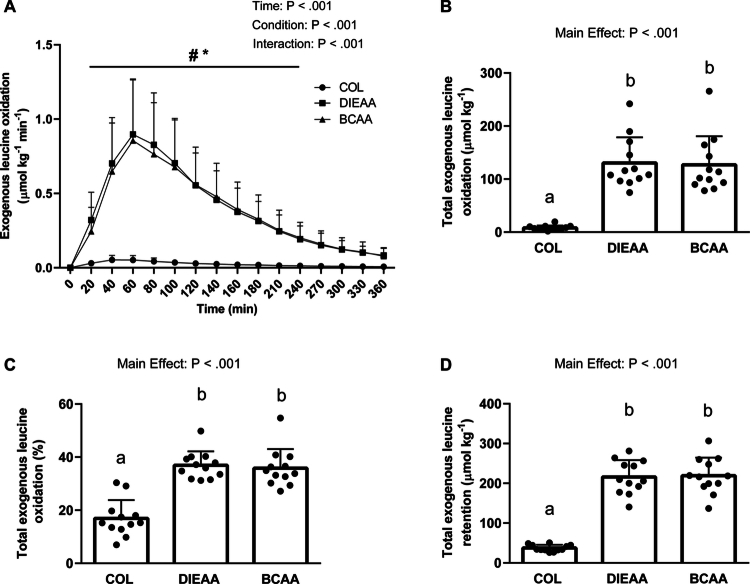
Exogenous leucine oxidation over the 6 h postprandial period (A), where ^#^denotes a significant difference between DIEAA and COL and *denotes a significant difference between BCAA and COL. Total exogenous leucine oxidation is expressed as the area under the curve (B) or the percentage of total leucine intake (C). Total exogenous leucine letention area under the curve (D). *P* < 0.0001 for all main effects of condition, time, or condition×time interaction. The conditions that do not share a letter are significantly different (*P* < 0.0001). COL, Collagen; DIEAA, Dileucine-containing essential amino acid formula; BCAA, Branched-chain amino acids.

There were significant time, condition, and interaction effects for Exo Ox (Figure 3A; all *P* < 0.0001). Exo Ox was greater in both DIEAA and BCAA compared with COL between *t* = 20−240 min (*P* < 0.05), with no difference between DIEAA and BCAA. There was a significant main effect of supplement condition for total exogenous leucine oxidation (*P* < 0.0001; Figure 3B). Total exogenous leucine oxidation was similar between DIEAA (130.36 ± 48.24 μmol·kg^−1^) and BCAA (126.93 ± 53.74; *P* = 0.678), whereas DIEAA and BCAA were both greater than those of COL (7.98 ± 4.51; both *P* < 0.0001). When expressed as a percentage of the ingested leucine (Figure 3C), total exogenous leucine oxidation was similar between DIEAA (36.94% ± 5.23%) and BCAA (35.84% ± 7.21%; *P* = 0.583) and greater for DIEAA and BCAA than for COL (16.92% ± 6.92%; both *P* < 0.001). Leucine retention (leucine intake—total exogenous leucine oxidation; Figure 3D) was similar between DIEAA (215.72 ± 42.45 μmol·kg^−1^) and BCAA (219.15 ± 45.26; *P* = 0.678) and greater in DIEAA and BCAA compared with COL (37.35 ± 8.16; both *P* < 0.0001).

### 3.4 *Ex vivo* serum does not influence protein ubiquitination, or markers of cell anabolism

**Figure 4.**
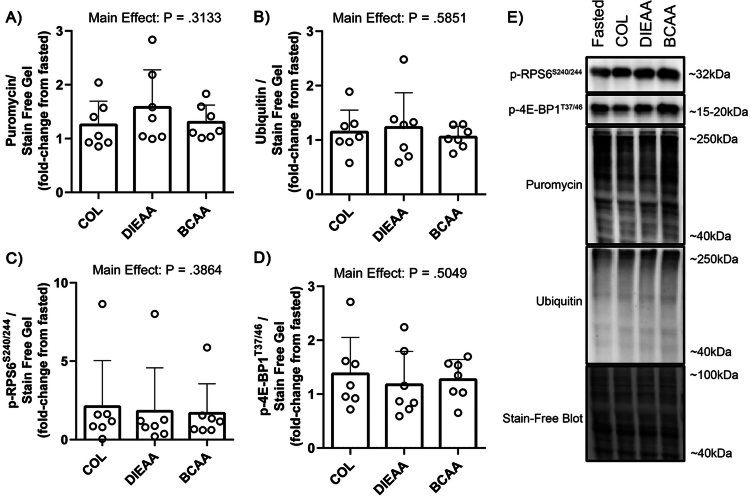
*Ex vivo* immunoblot analysis. Following 1 h of amino acid deprivation, C2C12 myotubes were treated with *ex vivo* human serum (20% v/v) in low-glucose amino acid-free DMEM for 4 h before collection and analysis via immunoblotting. Puromycin (1 µM) was added for the last 30 min of the serum period. A) Puromycin incorporation into nascent peptides. B) Protein ubiquitination. C) Phosphorylation of RPS6^S240/244^. D) Phosphorylation of 4E-BP1^T37/46^. E) Representative blots. (*n* = 7). COL, Collagen; DIEAA, Dileucine-containing essential amino acid formula; BCAA, Branched-chain amino acids.

**Figure 5.**
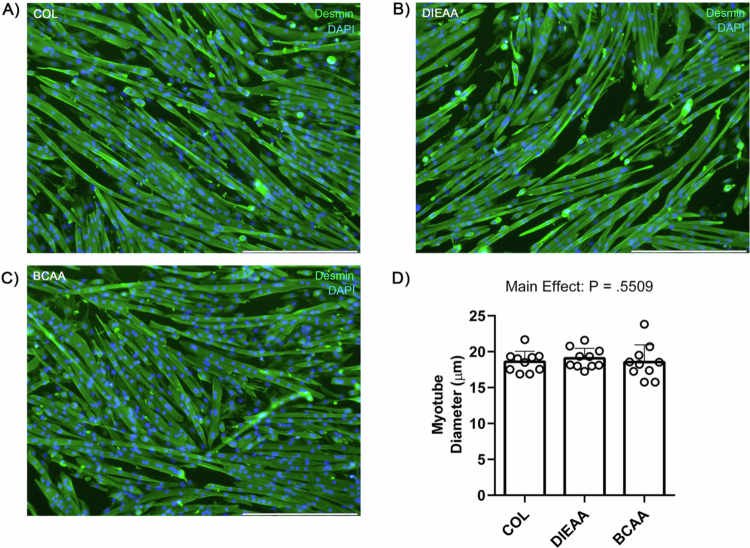
*Ex vivo* myotube diameter. Following 1 h of amino acid deprivation, C2C12 myotubes were treated with *ex vivo* human serum (20% v/v) in low-glucose amino acid-free DMEM for 4 h before being fixed and stained for desmin and DAPI. A–C) Representative images obtained with a 10× objective. D) Myotube diameter (*n* = 10). COL, Collagen; DIEAA, Dileucine-containing essential amino acid formula; BCAA, Branched-chain amino acids.

There was no effect of supplement condition *ex vivo* on puromycin incorporation into nascent peptides (*P* = 0.31; Figure 4A) or total protein ubiquitination (*P* = 0.59; Figure 4B), as determined by immunoblot. There was no effect of condition on the phosphorylation of the downstream mTORC1 substrates *P*-RPS6^S240/244^ (*P* = 0.39; Figure 4C) or *P*-4E-BP1^T37/46^ (*P* = 0.50; Figure 4D). Additionally, there was no effect of supplement condition on myotube diameter (*P* = 0.55; Figure 5D).

### 3.5 Dileucine-containing serum tends to stimulate protein synthesis *ex vivo*

**Figure 6.**
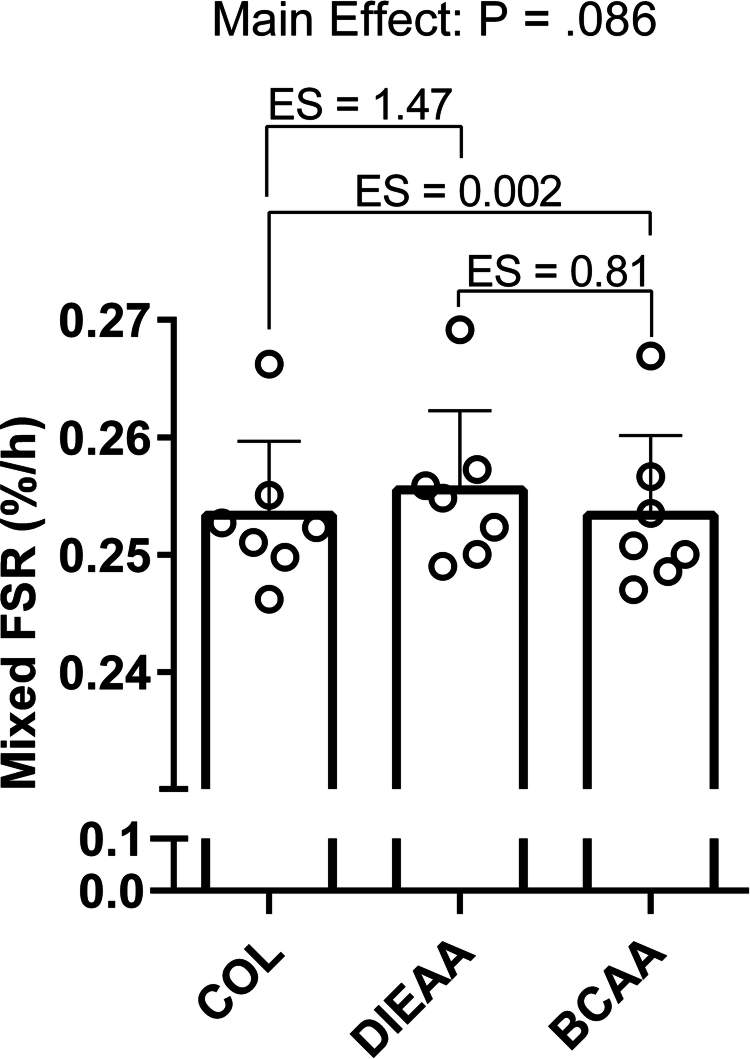
*Ex vivo* stable-isotope derived measurements of mixed-protein fractional synthetic rate (FSR) samples expressed in %/hour (*n* = 7). Following 1 h of amino acid deprivation, C2C12 myotubes were treated with *ex vivo* human serum (20% v/v) in low-glucose amino acid-free DMEM supplemented with 200 µM [^2^H_5_]phenylalanine for 4 h to measure protein synthesis. Effect sizes (Cohen's dz) are shown for each pairwise comparison. COL, Collagen; DIEAA, Dileucine-containing essential amino acid formula; BCAA, Branched-chain amino acids.

Mixed FSR (Figure 6) demonstrated a trend toward a main effect (*P* = 0.086). Pairwise comparisons revealed a large effect of DIEAA compared to COL (dz = 1.47 [95% CI: 0.08, 2.85]), a medium effect of DIEAA compared to BCAA (dz = 0.81 [–0.28, 1.89]), and a trivial effect of BCAA comapred to COL (dz = 0.002 [–0.92, 0.93]).

## 4 Discussion

The present study demonstrated that the ingestion of a dileucine-containing essential amino acid supplement stimulated total exogenous leucine oxidation and leucine retention, similar to leucine-matched BCAA supplementation, and BCAA and DIEAA increased leucine retention to a greater extent than did collagen protein control after whole-body resistance exercise. The differences in postexercise leucine kinetics were independent of changes in urinary 3MH:Cr, an indicator of myofibrillar protein breakdown. Furthermore, using an *ex vivo* cell model, we demonstrated that DIEAA had a greater effect on mixed muscle protein synthesis (measured by tracer incorporation) compared to COL, potentially due to the postprandial increase in EAA, leucine, and dileucine concentrations.

3MH, formed by the methylation of peptide-bound histidine in actin and myosin, is a nonproteogenic amino acid that is excreted in urine following protein breakdown [15]. Owing to the high 3MH content in skeletal muscle relative to other tissues [42], the urinary 3MH concentration has been used as a surrogate marker of skeletal muscle myofibrillar protein breakdown [43]. We have previously shown that higher daily EAA intake can attenuate plasma 3MH in response to endurance training [44]. We have also shown that 6 g of leucine-enriched EAA, which is expected to attenuate the normal resistance exercise-induced rise in tracer-derived rates of mixed muscle protein breakdown [4], reduces urinary 3MH relative to BCAA and carbohydrate-only ingestion after a bout of high-intensity, high-volume body weight resistance exercise [12]. In contrast to our hypothesis and these previous observations, there were no differences in urinary 3MH between any of the tested supplementation conditions in the present study. As we did not have a control, we are unable to determine whether our exercise stimulus elicited an increase in myofibrillar protein catabolism, which may have been similarly attenuated by all nutritional conditions. Alternatively, given that muscle protein breakdown may be elevated for up to 24 h during recovery from resistance exercise [45], our 6-h measurement period may not have captured the full postexercise catabolic response to dietary amino acid supplementation that other studies using EAA did over a 24-h measurement period [17]. Furthermore, the present study compared postexercise urinary 3MH with isonitrogenous amino acid/protein ingestion rather than amino acid-free 3MH [12] or nonnutritive fasted controls [17]. Consequently, we cannot discount the possibility that DIEAA or BCAA may attenuate urinary 3MH:Cr after resistance exercise relative to the fasted postexercise state if measured over a longer period.

Using repeated breath sampling in combination with an oral [1-^13^C]leucine tracer that is primarily metabolized within the lean tissues of the body [46], we assessed the post-exercise retention of dietary leucine as a proxy for whole-body protein synthesis [24]. Exogenous leucine oxidation (ExoOx; Figure 3A) peaked at 60 min for DIEAA and BCAA, which is in line with our previous work using crystalline EAA [12]. Moreover, exogenous leucine oxidation returned to baseline values by 360 min, suggesting that the 6-h measurement period was sufficient to recover the ^13^C tracer from the bicarbonate pool. Consistent with previous observations showing that postexercise leucine oxidation increases with increasing leucine intake [12,47], the present study demonstrated that total exogenous leucine oxidation was higher with DIEAA and BCAA compared to COL. Since the proportion of leucine oxidized was similar between DIEAA and BCAA, the total exogenous leucine retention in these groups was also similar, and both had greater total exogenous leucine retention compared to COL. The negligible effect of COL supplementation on postexercise leucine retention for whole-body anabolism is ultimately due to its limited essential amino acid and leucine contents [48]. While it has previously been shown that dileucine ingestion results in greater myofibrillar protein synthesis than does an equivalent dose of leucine in rested skeletal muscle [13], we did not observe any difference between DIEAA and BCAA at the whole-body level after exercise. As the leucine content of a meal impacts postprandial whole-body leucine oxidation and nonoxidative leucine disposal [49], the similar retention between DIEAA and BCAA may be related to the comparable leucine content and plasma leucinemia.

To further assess the anabolic potential of the nutritional beverages, we treated C2C12 myotubes with human serum-conditioned media as an *ex vivo* model of muscle anabolism [20]. There were no differences across conditions in myotube diameter, puromycin incorporation, or total protein ubiquitination, indicating that the treatments did not significantly impact muscle cell growth or the processes involved in protein degradation over the 4-h refeeding period. Moreover, there were no differences in the phosphorylation of the mTORC1 downstream signaling proteins *P*-RPS6^S240/244^ or *P*-4E-BP1^T37/46^ across conditions. Our results are in general contrast to those of previous studies demonstrating that media conditioned with serum from older adults fed complete protein at rest increased myotube hypertrophy [39]. The absence of differences between the effects of supplement conditions on mTORC1 substrate phosphorylation may be due to the serum in our study being obtained from young adults during the early postexercise period (<1 h) that is characterized by postexercise increases in anabolic hormones (e.g. testosterone, insulin-like growth factor 1, etc.) [50,51], which can support muscle growth and attenuate protein breakdown in this cell model [52,53] and therefore potentially minimize subtle nutrient-induced changes in hypertrophy. Additionally, we investigated the muscle protein synthetic response by measuring the incorporation of puromycin into immature peptides using the SUnSET technique [54] and into mature proteins via stable isotope incorporation (i.e. FSR) over the 4-h refeeding period. There were no statistical differences in puromycin incorporation across all conditions despite ~25% higher puromycin incorporation in DIEAA compraed to COL and BCAA. The inability to detect statistical differences with puromycin incorporation may be due to variability in the method, as previous work has also observed [39]. Utilization of puromycin for measuring protein synthesis is also limited in that puromycin is only added to the culture media for 30 min prior to cell collection and only serves as a proxy measurement, as puromycin incorporation into nascent peptides truncates their synthesis, resulting in nonfunctional peptides. Conversely, the direct measurement of MPS utilizing a stable isotope tracer over the entire 4-h serum-stimulation period demonstrated a large and medium effect for DIEAA compared to COL and BCAA, respectively, with a trivial effect of BCAA compared to COL. As leucine (and other EAA, to a lesser extent) have been demonstrated to be potent activators of mTORC1 and protein synthesis *in vitro* [6] and *in vivo* [55], the greater capacity of serum from the DIEAA condition to stimulate MPS (as measured with a tracer) compared to the COL condition is likely due to the increased substrate availability of EAA, as evidenced by serum concentrations (Figure 2). Overall, while confirmatory experiments with larger sample sizes and a true control are needed, our minimally invasive *ex vivo* approach suggests that DIEAA-containing serum may have an anabolic effect when supplemented into amino acid-free media at a 20% concentration. These experiments provide proof-of-principle to explore muscle anabolism *in vivo* in the future.

Notably, BCAA resulted in postprandial dileucine concentrations that were similar to those observed in DIEAA, despite lacking any dileucine in its formulation. This finding could suggest that BCAA ingestion stimulates endogenous dileucine production, albeit with a high degree of interindividual variability and a low absolute serum concentration. This effect may reflect a bioactive property of leucine, as previous research has shown that 2 g of leucine induces a subtle increase in plasma dileucine concentrations above fasting levels within the first 60 min after ingestion [13]. Thus, the potential that dileucine may be synthesized endogenously from ingested leucine and that it has important anabolic bioactivity may warrant further investigation.

In contrast to our hypothesis, we demonstrated that DIEAA stimulated postexercise whole-body protein anabolism to a similar extent as BCAA. While previous research has indicated that 2 g of dileucine can enhance myofibrillar protein synthesis more than 2 g of leucine alone at rest [13], our study showed no significant difference in postexercise whole-body protein anabolism between DIEAA and BCAA. This discrepancy may be attributed to the distinct differences between whole-body protein dynamics and localized myofibillar protein synthesis [56–59]. While BCAAs can stimulate myofibrillar protein synthesis [11], previous work suggests that the presence of other all the EAAs maximizes the magnitude and duration of myofibrillar protein synthesis to a greater extent than BCAAs alone [19,60]. Our *ex vivo* data further support this notion, showing a greater effect of DIEAA compared to COL for mixed muscle protein synthesis, suggesting that the additional EAAs support newly synthesized proteins at the muscle level. Considering the amino acid-induced stimulation of protein synthesis in an *ex vivo* model [20] is muted relative to the robust *in vivo* myofibrillar protein synthesis [32,33], *ex vivo* trends highlight the future need to examine how DIEAAs can support post-exercise muscle protein synthesis in humans*.*

In conclusion, dileucine-enriched essential amino acids and branched-chain amino acids supported greater leucine retention for whole-body anabolism compared with COL after resistance exercise independent of attenuation in estimates of myofibrillar protein breakdown. Moreover, our *ex vivo* experiments demonstrated a potential anabolic role of dileucine-enriched essential amino acids in stimulating MPS compared to a collagen-based amino acid composition. Collectively, our findings suggest that consuming dileucine within an EAA supplement is an effective strategy to support leucine retention for whole-body anabolism during recovery from resistance exercise in trained athletes.
